# Polysaccharides From Lentinus Edodes Inhibits Lymphangiogenesis *via* the Toll-Like Receptor 4/JNK Pathway of Cancer-Associated Fibroblasts

**DOI:** 10.3389/fonc.2020.547683

**Published:** 2021-02-12

**Authors:** Yuanyuan Wang, Yanqiong Zeng, Linyu Zhu, Jiajia Wan, Ningjing Lei, Xiaohan Yao, Xixi Duan, Yana Zhang, Yanru Cheng, Ning Tao, Zhihai Qin

**Affiliations:** ^1^ The First Affiliated Hospital of Zhengzhou University, Zhengzhou, China; ^2^ School of Basic Medical Sciences of Southwest Medical University, Luzhou, China; ^3^ Key Laboratory of Protein and Peptide Pharmaceuticals, Institute of Biophysics, Chinese Academy of Sciences, Beijing, China

**Keywords:** colorectal cancer, lymphangiogenesis, cancer-associated fibroblasts, VEGF-C, Lentinus edodes polysaccharides (MPSSS)

## Abstract

Polysaccharides from *Lentinus edodes* (*L. edodes*) have been successfully used as adjuvant chemotherapy drug to treat lymphatic metastasis in some malignancies, such as colorectal cancer (CRC), lung cancer and gastric cancer. The CRC could metastasize *via* lymphatic vessels. Lymphatic metastasis is commonly thought to be the cause of poor prognosis of CRC. The mechanism of polysaccharides from *L. edodes* inhibiting lymphatic metastasis of CRC is still unclear. In this study, we explored how MPSSS, a novel polysaccharide component of *L. edodes*, influences lymphangiogenesis and lymph node metastasis. The results show that MPSSS can reduce lymphangiogenesis and lymphatic metastasis of CRC in mouse model. And combined with *in vitro* study, a likely mechanism is that MPSSS reduce the secretion of VEGF-C by cancer associated fibroblasts (CAFs). This effect can be suppressed by a TLR4 inhibitor, which suggests that MPSSS plays a role in CAFs through the TLR4/JNK signaling pathway. In conclusion, MPSSS may reduce lymphangiogenesis by decreasing the VEGF-C secretion of CAFs, which may provide a new strategy for the comprehensive treatment of CRC.

## Introduction

The morbidity and mortality of CRC are both high throughout the world. CRC is the third most common malignant tumor and one of the leading causes of cancer-related mortality globally according to epidemiological studies (1). Lymphatic metastasis is one of the main route of CRC metastasis (2, 3). It is considered to be a poor prognostic factor in CRC (4). Therefore, preventing lymphangiogenesis may be an important means to improving the curative effect and prognosis of CRC patients.

Lymphangiogenesis is a precondition of cancer cells metastasis to draining lymph nodes via lymphatic vessels, which is also a common step in tumor spreading (5–7). Lymphangiogenesis includes proliferation, sprouting, migration and tube formation. Sprouting is associated with the proliferation and migration of lymphatic vessels, which has been observed in various human cancers. The major factors that regulate lymphangiogenesis are VEGF-C/VEGF-D and their receptor VEGFR3 (8, 9). Previous studies have shown that lymphangiogenesis is a high risk factor of metastasis and poor prognosis for some tumors, such as melanoma, breast cancer and CRC (8, 10, 11). Chemotherapy is one of the most important methods of tumor treatment. However, reports have shown that in addition to inhibiting tumor growth, chemotherapeutic drugs can also affect the distant metastasis of tumors by increasing lymphangiogenesis and inducing lymphatic metastasis (12).

It has long been considered that the progression of tumors is closely related to the abilities of malignant cells such as proliferation, invasion, and metastasis. However, it has been demonstrated that the tumor microenvironment plays a key role in tumor progression (13). The tumor microenvironment consists of immune cells, endothelial cells and cancer-associated fibroblasts (CAFs) (14). CAFs are resident cells in the tumor microenvironment. They induce tumor growth, angiogenesis, inflammation, and metastasis by secreting a variety of cytokines (15). CAFs may promote lymphangiogenesis by secreting VEGF-C (16, 17). Studies have shown that CAFs have a significant impact on the occurrence and development of tumors, and these findings have certain implications for tumor treatment (18). It has also been found that CAFs are the first cells to generate VEGF-C in tumor model of mouse (19).

Polysaccharides extracted from *L. edodes* have been used to improve health for centuries in Asia, and Lentinan is one of which have been used as an adjuvant of chemotherapy in clinics to inhibit lymphatic metastasis of some tumors, such as bladder cancer, CRC and lung cancer, in recent periods (20–22). MPSSS is a novel polysaccharide from *L. edodes* that is different from lentinan. They are different in molecular formula and weight. In previous studies, we found that MPSSS has high degree of purity, it could decrease the immunosuppressive functions of myeloid-derived suppressor cells (MDSCs) and alter the function of prostate CAFs (13, 23). Whether MPSSS can inhibit lymphatic metastasis of tumors and the mechanism is poorly understood. In this study, we observed that MPSSS inhibited the VEGF-C secretion of CAFs *via* the TLR4/JNK pathway, thus reducing lymphangiogenesis and lymphatic metastasis of CRC in a mouse model.

## Materials and Methods

### Cell Culture and Cell Lines

SVEC4-10 cell line was purchased from ATCC (USA). CT-26 cell line was provided by Yan Li of Academy of Military Sciences PLA China.

A model of CAF was constructed from mouse embryonic fibroblasts (MEFs) based on our previous study (16). MEFs were transfected with hTERT retrovirus containing a puromycin-resistance gene. Immortalized MEFs with puromycin-resistance were co-injected with CT-26 cells to build tumor bearing mouse model. Tumor was dissociated after 10 days tumor growth and 2 μg/ml puromycin were used as selection (**Supplementary Figure 1**). MEFs upon co-injection with tumor cells mainly differentiated into CAFs (24).

TLR 4 (Toll-like receptor 4) + and TLR4 - MEFs were isolated from C57/BL6 and Toll-like receptor 4-/- (TLR4-/-) mice respectively.

CT-26-GFP cell line was constructed as follows. First, the GFP-PLPCX plasmid was constructed and transformed into competent DH5a. Then, the clones were sequenced correctly. The amplified

GFP-PLPCX plasmid and PCL-10A1 plasmid were co transfected into 293FT cells at the ratio of 1:1. After 60 h, the supernatant was collected and the cell debris was filtered out with a 0.45μm filter. The filtrate was the original virus solution. After 48 h, the tumor cells with GFP positive could be seen under fluorescence microscope. The GFP positive CT-26 cells could be obtained by flow cytometry.

All cells and cell lines were cultured in DMEM (HyClone, USA) with 10% fetal bovine serum

(PAN, Germany) and 1% penicillin-streptomycin (Beijing solarbio science & technology co., Itd, China) under the condition of 37°C and 5% CO_2_.

### Purification of MPSSS

The powder of *L. edodes* (Johncan International, China) was dissolved in deionized water and stirred overnight. After centrifugation, the supernatant was collected and mixed with anhydrous ethanol at a ratio of 1:1, and then the mixture was stored overnight in a refrigerator at 4°C. The precipitates were collected and dissolved in 30% (V/V) ethanol solution after centrifugation again. After centrifugation, anhydrous ethanol was added to the supernatant to form a 40% (V/V) ethanol solution. Twelve h later, the solution was similarly treated to obtain a 50% (V/V) ethanol solution. The precipitate was collected when the concentration of ethanol reached 80% (V/V). Finally, the precipitate was washed and dried with anhydrous ethanol and ether, and the resulting gray powder was MPSSS. MPSSS could easily soluble in any aqueous solution.1 ml DMEM was added to dissolve 1 mg MPSSS powder to obtain 1 mg/ml mother liquor for experiment *in vitro*. 1 ml PBS was added to dissolve 3 mg MPSSS powder to obtain 3 mg/ml mother liquor for experiment *in vivo*.

### Mouse Tumor Model

The BALB/c and C57/BL6 mice used in this study were purchased from Vital River Laboratories (Beijing, China). Toll-like receptor 4-/- (TLR4-/-) mice were purchased from Model Animal Research Center of Nanjing University.

To study the effect of MPSSS on lymphangiogenesis of CRC, total of 1 × 10^6^ CT-26 tumor cells (100μL) were injected subcutaneously into the flanks of BALB/c mice to build subcutaneous mouse model. Experiment group treated with MPSSS (30 mg/kg/day) and control group with PBS every day right after tumor cells planted. Tumor growth was monitored every other day after the palpable nodule appeared, and tumor volume was calculated by 1/2(length × width^2^). All mice were sacrificed on the 21th day after tumor implantation. Bloods of mice were collected from orbital venous plexus with glass capillary. Pressed both sides of the neck of the mice, causing congestion of the retroorbital venous plexus. The glass capillary and the mouse face formed an angle of 45 degree, and penetrated from the inner corner of the eye. The penetration depth is about 2–3mm. Tumors were also collected after the euthanize was performed for future use.

A total of 2 × 10^5^ CT26-GFP cells (25μL) were injected subcutaneously into the foot pad of mice to build footpad metastasis model to see the effect of MPSSS on metastasis of CRC. To fix the cells in a certain position, the cells suspension was mixed with Matrigel in an equal volume 1:1 (ml/ml). MPSSS (30 mg/kg/day) or PBS was injected intraperitoneally once a day. Draining inguinal lymph nodes (diLNs), draining posterior fossa lymph nodes (dpLNs) and tumors of all mice were harvested at the same time 25 days after tumor implantation. DiLNs and dpLNs were draining lymph nodes obtained from the same side which tumor cells planted.

To further investigate the mechanism of MPSSS *in vivo*, 1 × 10^5^ CT26-GFP cells were co-injected with TLR4+ and TLR4- mouse embryonic fibroblasts (MEFs) in a ratio of 1:3 (total volume 25μL) subcutaneously into the foot pad of mice to see whether MPSSS affected lymphangiogenesis through CAFs *in vivo*. Both groups of mice were treated with MPSSS (30 mg/kg/day). Then all tissues needed were harvested at the same time after the euthanize was performed.

### Immunofluorescence

Frozen sections of tumor tissues were fixed with 4% paraformaldehyde for 15 minutes and washed with PBS three times. The sections were blocked with 3% BSA for 30 minutes at room temperature. Then, the sections were incubated with Lymphatic Vessel Endothelial Hyaluronan Receptor-1 (LYVE-1) antibody (Abcam company, England) at 4°C overnight. All sections were washed with PBS three times followed by incubation with secondary fluorescently labeled antibody (Proteintech, USA). Mounted sections with mounting media containing DAPI (Electron Microscopy Sciences, USA). The control group incubated with secondary antibody only. Photos were taken by Vectra (PerkinElmer, USA).

### Flow Cytometry

Flow cytometry was performed to test the tumor cells migrated to draining lymph nodes, the diLNs and dpLNs were removed from footpad tumor-bearing mice after the euthanize was performed. Then, all lymph nodes were ground separately and digested with collagenase in a 37°C incubator for 20 minutes. The concentration of collagenase was 1 mg/ml. Collagenase was neutralized with DMEM media, and the liquid with lymph node tissue was filtered. 5 × 10^5^ cells from each cell suspension were stained with 7-amino-actinomycin D (7-ADD, 0.25 ^µg/105^ cells, BD Bioscience) to eliminate dead cells before flow cytometry. Equal amounts of cells (50,000) were harvested from the suspension of each sample by FACS (Becton, Dickinson and Company, USA). The GFP positive cells were tumor cells migrated from tumor tissues to lymph nodes. The percentage of GFP positive cells may represent the metastasis of tumor.

### Cell Viability Assay: Cell Counting Kit-8 Assay

Cell Counting Kit-8 (CCK-8) was used to evaluate MPSSS-induced cytotoxicity. The SVEC4-10 cell line was used as lymphatic endothelial cells in this study. CT-26 CAFs and SVEC4-10 cells were seeded into 96-well plates at a density of 3,000 cells/well, and incubated with MPSSS at different concentrations for 24 h. 5-Fluorouracil (5-FU) (MedChemExpress, USA) of different concentrations (0-10 µg/ml) were also used to treat CAFs as positive control. Then, 10 µL of the CCK-8 reagent was added to each well and incubated for 2 h. The absorption at 450 nm was measured with a microplate reader. To detect the effect of MPSSS-treated CAF supernatants on the proliferation of SVEC4-10 cells, SVEC4-10 cells were cultured in 96-well plates at a density of 3,000 cells/well, and treated with media contained 30% CAF supernatants. CAFs were seeded in 6-well plates at a density of 5 × 10^5^ cells/well. Different treatments were added after the cells adhered to the plates. After 24 h of stimulation, supernatant was collected and centrifuged. CAFs treated with 30 µg/ml MPSSS or inhibitors were used as the experimental group, and PBS was used as the negative control.

### Scratch Wound Healing Assay

SVEC4-10 cells were seeded in 6-well plates at a density of 5 × 10^5^ cells/well. After the wells were full of cells, fibronectin and poly-L-lysine were added to cover the cells. Then a straight line was drawn to create a scratch in each well, and the media was exchanged with 30% CAF supernatant, while the control group media was exchanged with DMEM complete media containing 30% FBS free DMEM. Then, photos were taken at different time points (0, 4, 8, and 12 h). The gap areas were calculated with ImageJ software. This is double-blind experiment to avoid bias.

### Transwell Assay

Media containing 30% CAF supernatant (experimental group) and 30% FBS free DMEM (control group) was added to the 24-well plate. Media with 5 × 10^5^ SVEC4-10 cells were added to the upper chamber. After incubation in a 37°C incubator for 4 h, the non-migrated cells in the upper chamber were wiped off and dyed in crystal violet for 15 minutes. Then, the chambers were washed with PBS three times, and photos were taken under a microscope.

### Real-Time PCR

RNA samples were extracted from CT-26 CAFs and tumor tissues of mouse models. CT-26 CAFs were seeded in 6-well plates at a density of 5 × 10^5^ cells/well and treated with MPSSS at 30 µg/ml for different times (0 h, 12 h). Tumor tissues of equal weights tumor tissues were treated with liquid nitrogen and ground. Total RNA was extracted from cells and tumor tissues with TRIzol

(Invitrogen, Carlsbad, CA, USA) and quantified on an ND-1000 spectrophotometer. The extracted mRNA was synthesized into the first chain cDNA using a Primescrip RT reagent kit

(TaKaRa, Tokyo, Japan). Real-time PCR was carried out using SYBR Premix ExTaq

(TaKaRa, Tokyo, Japan) according to the manufacturer’s instructions. GAPDH mRNA was used as an internal control. The specific primers were as follows: vegfc: 5’- GAGGTCAAGGCTTTTGAAGGC-3’; 5’- CTGTCCTGGTATTGAGGGTGG-3’; gapdh: 5’- AGGTCGGTGTGAACGGATTTG-3’; and 5’- TGTAGACCATGTAGTTGAGGTCA-3’. Primers were purchased from Sangon Biotech Shanghai Co., Ltd.

### Western Blot Assay

CT-26 CAFs were seeded in six-well plates at a density of 5 × 10^5^ cells/well and treated with different concentrations of MPSSS (0, 20, and 50 µg/ml) for 3 h and then lysed with RIPA buffer supplemented with 100 mM phenylmethylsulfonylfluoride, 25 µg/ml aprotinin, 1 mM sodium orthovanadate and 50 nMNaF to obtain total protein. A BCA protein assay was used to determine the protein concentrations. Aliquots of protein samples were resolved on a 10% SDS-PAGE gel and then transferred to a nitrocellulose membrane (GE Healthcare, Milwaukee, WI, USA) using a semi-dry transfer apparatus (Bio-Rad Laboratories). Then, the membrane was blocked with 5% skim milk in PBST (0.1% Tween-20) for 2 h and incubated overnight at 4°C with primary antibodies. The primary antibodies used were against JNK, p-JNK, phospho-NF-ĸB p65, and NF-ĸB p65 (all from Cell Signaling Technology, USA) were used, all of which were diluted at 1:1,000, and antibody against GAPDH (Abclonal Biotechnology, USA) at 1:4,000 was used as an internal standard.

### ELISA

This assay was conducted according to the manufacturer’s instructions using ELISA kits (Reddot Biotech Inc., Canada). The samples were subjected in triplicate. Absorbance at 450 nm was measured with an automated microplate reader. The concentrations of VEGF-C were calculated according to the standard curve provided by ELISA kit.

### Statistical Analysis

Data are expressed as the mean ± SD of at least three independent experiments. Statistical significance was calculated using Prism 6.0 Graphpad software. Student’s t test was used to make a single comparison between the two groups. For experiments including more groups an ANOVA multivariate analysis was performed accompanied by a post-hoc modification test. P values <0.05 were considered significant.

## Results

### MPSSS Reduces Lymphangiogenesis in a Subcutaneous Tumor-Bearing Mouse Model

**Figure 1:**
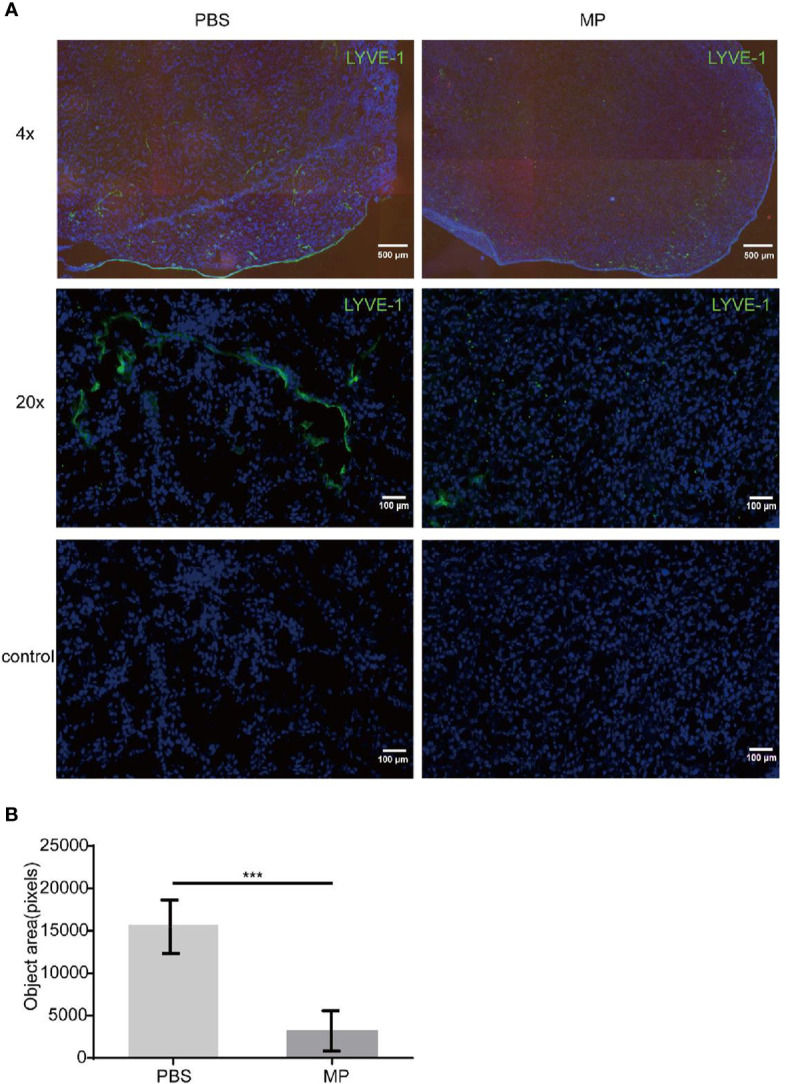
MPSSS inhibits lymphangiogenesis in tumor-bearing mice. CRC tumor-bearing mice were injected with PBS and MPSSS (30 µg/kg) intraperitoneal every day. Vectra was used to take photos of sections after IF staining **(A)**. The fluorescence intensity of LYVE-1 was represented by pixels with InForm software **(B)**. The data were recorded as the mean ± SD and analyzed by t-test. MP is short for MPSSS. (***p < 0.001).

The mice were sacrificed on the 21^th^ day of tumor implantation. Lymphatic vessels in tumor tissues were marked with LYVE-1 by immunofluorescence. LYVE-1 is a relatively specific marker of lymphatic vessels. The number of lymphatic vessels in the sections was quantified and calculated by inForm software of Vectra. The software could measure all fluorescent region and record these areas as pixels. There were six mice in each group and more than three photos were taken of each section. Statistical analysis of the data was performed by GraphPad Prism version. The expression of LYVE-1 in the PBS group was higher than that in the MPSSS group (**Figure 1A**). The lymphatics were continuous and complete in the PBS group, while complete lymphatics could barely be seen in the MPSSS group. The number of lymphatics in the PBS group was greater than that in the MPSSS group according to statistical analysis, and the difference was statistically significant (**Figure 1B**).

### MPSSS Reduces Lymphatic Metastasis of CT-26 Tumors in a Footpad Tumor-Bearing Mouse Model

**Figure 2:**
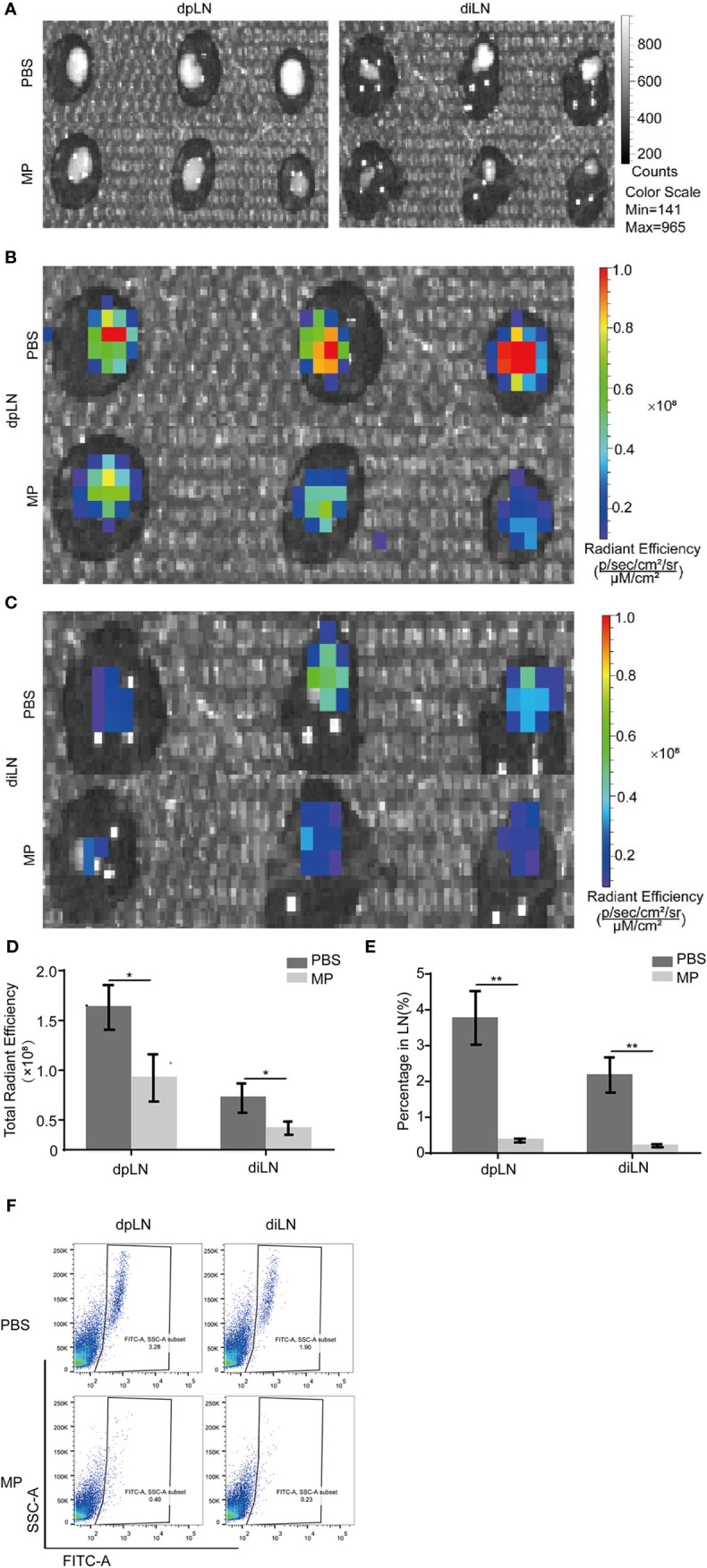
MPSSS Reduces CRC metastasis in a mouse model. CT-26-GFP cells were injected subcutaneously into the footpads of BALB/c mice. Draining lymph nodes different groups were harvested at the same time after cervical dislocation. Draining lymph nodes were taken photos by Caliper IVIS Lumina II under bright field **(A)** and fluorescent field **(B, C)**. Fluorescence intensity was quantified and analyzed **(D)**. Then, all lymph nodes were ground separately and digested with collagenase. Flow cytometry analysis was shown to display the percentage of GFP (+) cells migrating to draining lymph nodes **(E, F)**. Data were analyzed by t-test. (*p < 0.05. **p < 0.01).

A footpad tumor-bearing mouse model was established to observe the effect of MPSSS on tumor lymphatic metastasis. It is an appropriate model for observing lymphatic metastasis because mouse footpads have abundant lymphatic vessels. Mice in each group were treated with MPSSS (30 µg/kg) or PBS. There were six mice in each group. CT-26 cells injected in the footpads were labeled with GFP. The dpLNs and diLNs in each group of mice were collected after the euthanize was performed on the 25^th^ day after tumor implantation. Pictures were taken by Caliper IVIS Lumina II under bright field (**Figure 2A**) and fluorescent field (**Figures 2B, C**). The fluorescence intensity of lymph nodes could represent the number of GFP positive cells migrated to lymph nodes. The fluorescence intensity of dpLNs from PBS -treated mice was stronger than that from MPSSS -treated mice (**Figure 2B**). The same phenomenon was observed in diLNs (**Figure 2C**). The statistical results of radiant efficiency analysis indicated that MPSSS inhibited the lymphatic metastasis of CRC (**Figure 2D**). Next, all the lymph nodes were separately ground and treated in the PBS group than in the MPSSS group in both diLNs and dpLNs (**Figures 2E, F**). These results suggest that MPSSS inhibits CRC metastasis in the mouse model.

### MPSSS Inhibits the Proliferation of SVEC4-10 Cells Through CT-26 CAFs

To investigate the mechanism by which MPSSS affects lymphangiogenesis, a CCK-8 assay was used to detect the direct and indirect effects of MPSSS on the proliferation of SVEC4-10 cells.

**Figure 3:**
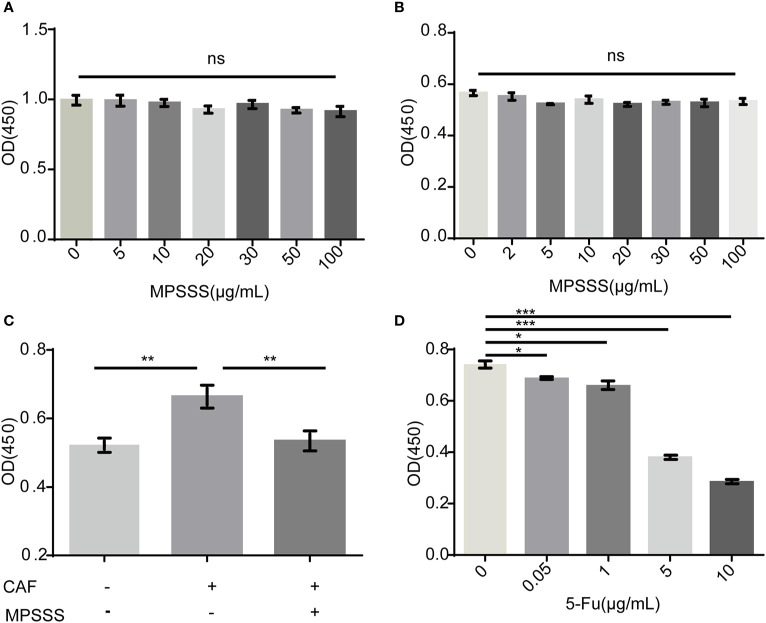
MPSSS inhibits the proliferation of SVEC4-10 cells through CAFs. Different concentrations of MPSSS were used to treat SVEC4-10 cells **(A)** and CT-26 CAFs **(B)** for 24 h. SVEC4-10 cells were incubated with 30% CAF supernatant treated with MPSSS **(C)**. Different concentrations of 5-Fu (0–10 µg/ml) were used to treat CT-26 CAFs as positive control **(D)**. Cell viability was measured at OD450. The proliferation of cells was expressed as the mean ± SD from triplicate measurements and analyzed by one-way ANOVA and t-test. (ns p>0.05. *p < 0.05. **p < 0.01. ***p < 0.001). ns represents not statistically significant.

SVEC4-10 cells incubated with CAF supernatant were treated with 30 µg/ml MPSSS or FBS free DMEM (negative control). The results showed that different concentrations of MPSSS (0-100 µg/ml) had no direct effect on the proliferation of SVEC4-10 (**Figure 3A**) and CT-26 CAFs (**Figure 3B**). While CAF supernatant promoted the proliferation of SVEC4-10 cells, supernatant treated with MPSSS inhibited the proliferative effect of CAF supernatant on SVEC4-10 cells (**Figure 3C**), which indicated that MPSSS inhibited the proliferation of SVEC4-10 cells *via* CAFs. 5-Fluorouracil was used as positive control (**Figure 3D**).

## MPSSS Inhibits the Migration of SVEC4-10 Cells *via* CT-26 Cancer Associated Fibroblasts

**Figure 4:**
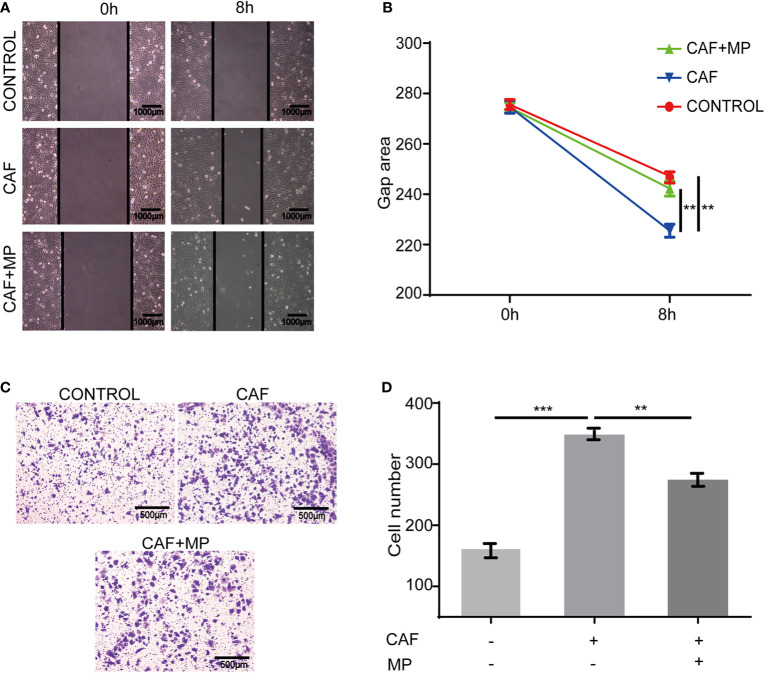
The effect of MPSSS on the migration of SVEC4-10. 30% CAF supernatant treated with MPSSS (30 µg/ml) was used to culture SVEC4-10 cells. Photos were taken with a camera under a microscope at different time points (0 and 8 h) **(A, B)**. Thirty percent CAF supernatant treated with and without MPSSS (30 µg/ml) was added to the bottom chamber **(C, D)**. Gap area and cell number were expressed as the mean ± SD from triplicate measurements and analyzed by one-way ANOVA and t-test. (**p < 0.01. ***p < 0.001).

In a real tumor environment, lymphatic endothelial cells need to migrate to cells that can secrete VEGF-C before proliferation. Therefore, scratch and Transwell assays were used to investigate the effect of MPSSS on the migration of SVEC4-10 cells. The gap area of SVEC4-10 cells treated with CAF supernatant was smaller than that of cells treated with media. MPSSS reversed this effect of CAF supernatant (**Figures 4A, B**). More SVEC4-10 cells migrated to the lower chamber in the CAF supernatant -treated group than in the DMEM (free of FBS) -treated group. MPSSS -treated CAF supernatants decreased the number of migrated cells (**Figures 4C, D**). Both results demonstrated that MPSSS inhibited the migration of SVEC4-10 cells via CAFs.

### MPSSS Inhibits the Proliferation and Migration of SVEC4-10 Cells Through the Toll-Like Receptor 4/JNK Pathway in Cancer Associated Fibroblasts

**Figure 5:**
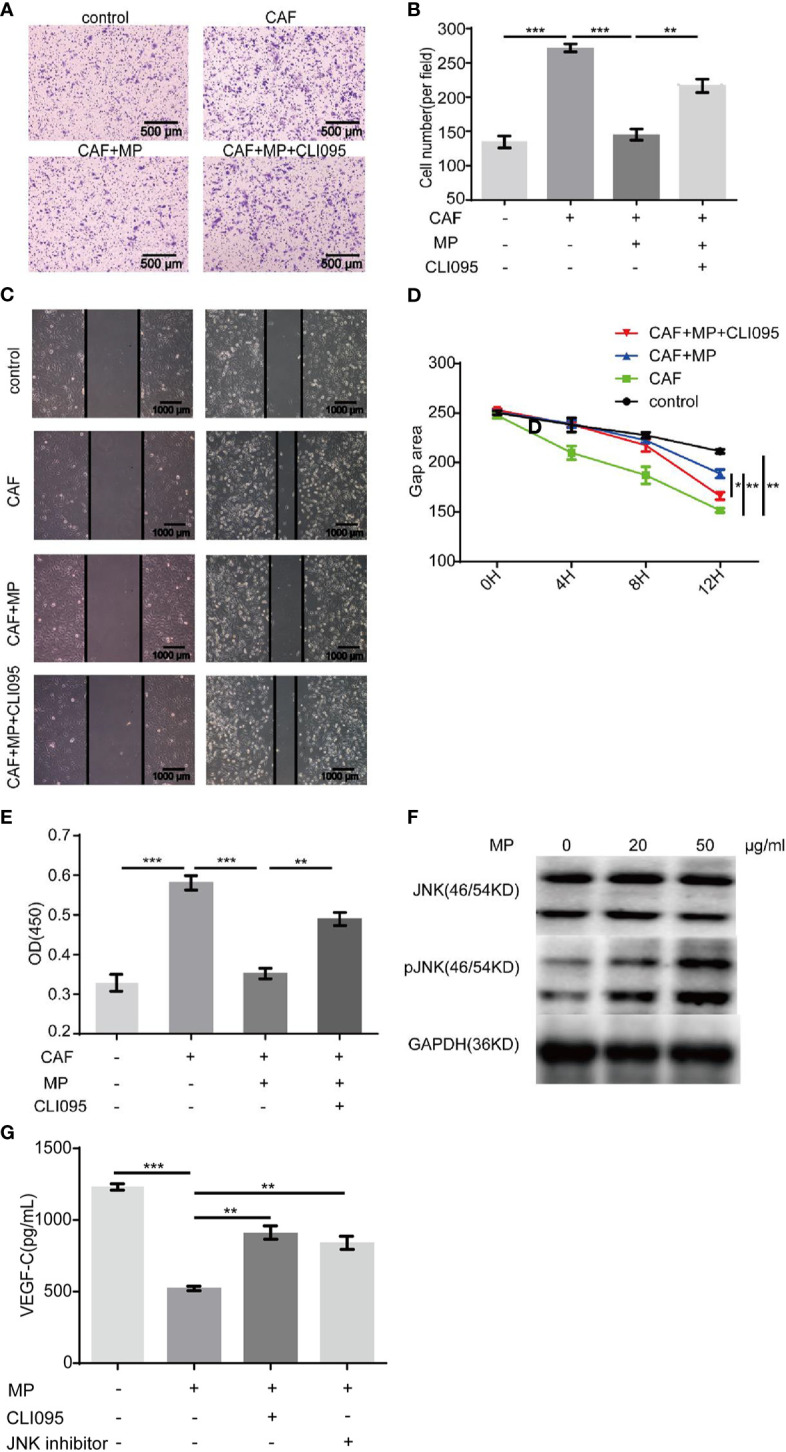
CLI-095 weakened the effect of MPSSS on SVEC4-10 cells. Media containing thirty percent CAF supernatant treated with MPSSS (30 µg/ml) or CLI-095 was added to the bottom Transwell chambers. Media containing thirty percent DMEM (without FBS) was negative control. The non-migrated cells were wiped off after 4 h. The migrated cells were photographed **(A, B)**. Thirty percent CAF supernatant treated with MPSSS (30 µg/ml) or CLI-095 and DMEM (without FBS) (negative control) was used to culture SVEC4-10 cells. Photos were taken with camera under microscope at different time points (0, 4, 8, and 12 h) **(C, D)**. Similarly, SVEC4-10 cells were cultured with 30% CAF supernatants treated with MPSSS (30 µg/ml) or CLI-095 for 24 h. Cell viability was measured at OD450 **(E)**. CAFs were incubated with MPSSS (0, 20, and 50 µg/ml) for 3 h. Total protein was collected, and protein levels were analyzed by Western blotting **(F)**. CAFs treated with MPSSS, CLI-095 and JNK inhibitor for 24 h. The supernatant was collected and measured with ELISA kit to determine the concentration of VEGF-C **(G)**. Data were expressed as the mean ± SD from triplicate measurements and analyzed by one-way ANOVA. (*p < 0.05. **p < 0.01. ***p < 0.001).

MPSSS is a polysaccharide, and TLR4 is a polysaccharide receptor. We treated CAFs with the TLR4 inhibitor CLI-095 (MedChemExpress, USA), and the supernatant was collected and cultured with SVEC4-10 cells to observe its effect. The concentration of CLI-095 was 2μM based on our previous study (13). Scratch tests and Transwell assays both showed that CLI-095 weakened the migration-inhibiting effect of CAF supernatant (treated with MPSSS) on SVEC4-10 cells (**Figures 5A–D**). CLI-095 also attenuated MPSSS-induced proliferation inhibition in SVEC4-10 cells (**Figure 5E**). Western blot analysis was performed to identify the pathway associated with the MPSSS effect. The results showed that MPSSS increased the phosphorylation of JNK (**Figure 5F**). To further investigate the effect of JNK, CAFs were treated with a JNK inhibitor, SP600125 (TargetMol, USA).The concentration was 10μM. The VEGF-C expression was measured by ELISA. ELISA assay showed that MPSSS inhibited the VEGF-C secretion of CAFs, while CLI-095 and JNK inhibitor attenuated this effect (**Figure 5G**). We found that TLR4 is the receptor of MPSSS on CT26 CAFs, and that MPSSS regulates the secretion of VEGF-C through the TLR4/JNK pathway.

### MPSSS Reduces VEGF-C Secretion Both *In Vivo* and *In Vitro*

**Figure 6:**
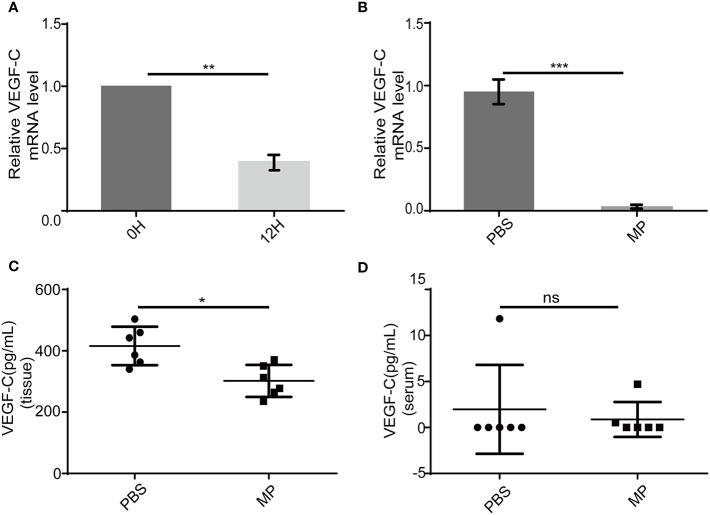
MPSSS decreases VEGF-C production both *in vitro* and *in vivo*. After incubation with MPSSS (30 µg/ml) for 12 h, the total RNA was extracted from CAFs for real-time PCR **(A)**. Tumor tissues of mice were harvested after cervical dislocation. The same weight of each tumor was ground for real-time PCR **(B)** and ELISA **(C)**. Blood from each mouse was taken by capillary pipette for ELISA before the euthanize was performed **(D)**. Data of real-time PCR were analyzed by t-test. Data of ELISA were expressed as the mean ± SD. (ns p>0.05. *p < 0.05. **p < 0.01. ***p < 0.001). ns represents not statistically significant.

VEGF-C is a main factor that causes lymphangiogenesis. It is not clear whether MPSSS -mediated inhibition of SVEC4-10 proliferation is related to VEGF-C. We used PCR and ELISA to validate this hypothesis. VEGF-C produced by CAFs decreased after treatment with MPSSS (30 µg/ml) *in vitro* (**Figure 6A**). Tumor tissues were lysed with trizol after they were ground in liquid nitrogen. RNA was extracted for real-time PCR. The results showed that MPSSS decreased the expression of VEGF-C in tumor tissues (**Figure 6B**). ELISA assay results showed that the concentration of VEGF-C was higher in PBS -treated tumor tissues than in MPSSS -treated tumor tissues (**Figure 6C**). The concentration of VEGF-C in serum was also detected and there was no difference between the two groups (**Figure 6D**). This result indicated that the VEGF-C affecting lymphangiogenesis was derived from tumor tissue.

### MPSSS Reduces Lymphatic Metastasis *via* Toll-Like Receptor4 of Cancer Associated Fibroblasts *In Vivo*

**Figure 7:**
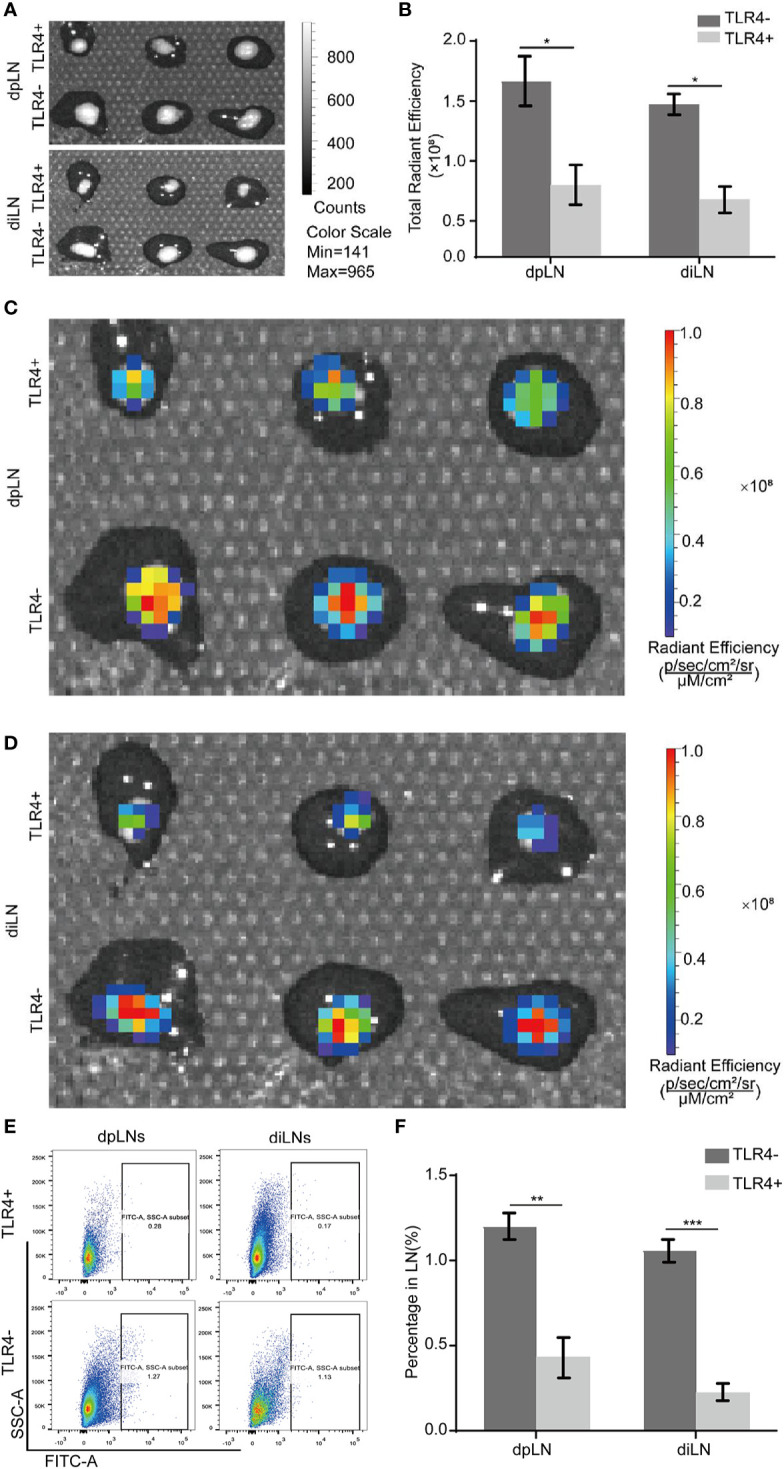
MPSSS reduces lymphatic metastasis *via* TLR4 of CAFs *in vivo*. CT-26-GFP cells were co-injected with MEFs (TLR4+ and TLR4-) subcutaneously into the footpads of WT mice. Draining lymph nodes were harvested and taken photos by Caliper IVIS Lumina II under bright field **(A)** and fluorescent field **(C, D)**. Fluorescence intensity of lymph nodes was quantified and analyzed **(B)**. The percentage of GFP (+) cells migrating to draining lymph nodes was shown by flow cytometry analysis **(E, F)**. Data were analyzed by t-test. (*p < 0.05, **p < 0.01, ***p < 0.001).

To investigate whether MPSSS reduced lymphatic metastasis *via* TLR4 of CAFs, CT-26 cells labeled with GFP were co-injected with TLR4+ MEFs and TLR4- MEFs subcutaneously into the foot pad of mice. There were six mice in each group. MPSSS was injected intraperitoneally every day in both group of mice. The dpLNs and diLNs were collected on the 25^th^ day after tumor implantation (**Figure 7A**). The fluorescence intensity of lymph nodes was detected by Caliper IVIS Lumina II. The fluorescence intensity of dpLNs and diLNs from TLR4- MEFs group mice was stronger than that TLR4+ MEFs group (**Figures 7C, D**). Statistical analysis of lymph nodes radiant efficiency analysis indicated that TLR4 on CAFs has a certain effect on MPSSS (**Figure 7B**). The percentage of cells expressing GFP was higher in the TLR4- group than in the TLR4+ group in both diLNs and dpLNs (**Figures 7E, F**). This result indicated that MPSSS may inhibit the lymphatic metastasis of CRC by TLR4 of CAFs.

## Discussion

In this study, we first demonstrated that MPSSS inhibits lymphangiogenesis and lymphatic metastasis through CAFs in CRC. The TLR4/JNK pathway of CAFs plays an important role in this process.

Lymphangiogenesis is a common phenomenon in the process of tumor occurrence and development, and is considered to be closely related to the poor prognosis of tumor patients (10). The discontinuous structure of the lymphatics determines that cells can easily enter the lymphatics through the monolayer lymphatic endothelium (5). Lymphangiogenesis is regulated by some cytokines and signaling pathways, and the increase in lymphangiogenesis may cause distant metastasis of solid tumors. Conventional treatment chemotherapy can treat tumors, but it can increase lymphangiogenesis and lead to lymphatic metastasis of tumors. This process is mainly realized *via* the VEGF-C/VEGFR3 axis (12). Therefore, reducing lymphangiogenesis requires inhibiting the secretion of VEGF-C in tumors, which may reduce lymphatic metastasis and improve the prognosis of tumor patients. We found that MPSSS could reduce lymphatic metastasis by inhibiting lymphangiogenesis in our study, but the structure and integrity of lymphatic vessels are also important factors of lymphatic metastasis. Whether MPSSS could affect the structure of lymphatic vessels is unknown. Other cytokines such as VEGF-D could also induce the formation of lymphatic vessels in tumor (6, 25), whether MPSSS could affect the VEGF-D secretion of CAFs and then induce lymphangiogenesis need further study.

In the tumor microenvironment, there are many kinds of cells that can secrete VEGF-C, such as CAFs, macrophages, tumor cells and even lymphatic endothelial cells. Of all these cells, CAFs are the resident cells and the very first cells to secret VEGF-C during tumorigenesis. Previous studies confirmed that MPSSS could act on CAFs and change their functions. Therefore, we chose CAFs as the research object. MPSSS might also affect lymphangiogenesis *via* other cells, but those were not involved in our study.

MPSSS is a new polysaccharide extracted from *L. edodes*. Our previous studies showed that MPSSS can inhibit tumor growth, change the inhibitory effect of CAFs on T cells, and reduce the immunosuppression of MDSCs (13, 23). Lentinan can be combined with chemotherapy drugs in clinical cases to reduce the lymphatic metastasis of malignant tumors to improve the prognosis of patients (26). MPSSS and Lentinan are both extracted from *L. edodes*, but whether they have same function is unknown. In this experiment, we confirmed that MPSSS can inhibit the secretion of VEGF-C by CAFs and decrease the concentration of VEGF-C in tumor tissue, thus reducing lymphangiogenesis and lymphatic metastasis of tumors.

In previous studies, we found that the receptor of MPSSS on CAFs is TLR4 (13), so we treated CAFs with CLI-095, an inhibitor of the TLR4 receptor, and collected supernatant to observe its effect on lymphatic endothelial cells. CLI-095 weakened the inhibitory effect of MPSSS on the proliferation and migration of lymphatic endothelial cells. WB results showed that the phosphorylation of JNK increased in CAFs treated with MPSSS, while CLI-095 inhibited this effect.

The model used in this study is a mouse model of CRC. Lymphangiogenesis is common in human CRC and closely related to tumor lymphatic metastasis. Patients with a high density of lymphatics have a poor prognosis and short survival time (11). Therefore, lymphangiogenesis is an important factor for evaluating the prognosis of CRC patients. Our experiment proved that MPSSS could inhibit lymphangiogenesis and reduce lymphatic metastasis in a mouse CRC model. There are other solid tumors that metastasize mainly through lymphatic vessels, the effects of MPSSS on these tumors need to be further investigated.

## Conclusion

In summary, this study demonstrated that MPSSS can inhibit lymphangiogenesis and lymphatic metastasis in CRC, which was achieved *via* the TLR4/JNK pathway of CAFs cells. Blocking the TLR4/JNK pathway may be a potential therapeutic target for CRC metastasis.

## Data Availability Statement

The original contributions presented in the study are included in the article/**Supplementary Material**, further inquiries can be directed to the corresponding authors.

## Ethics Statement

The animal study was reviewed and approved by the Biological Research Ethics Committee, Institute of Biophysics, Chinese Academy of Sciences.

## Author Contributions

YW is the first author, responsible for most of the experiments in this paper and the writing of the article. YZe, JW, XY, XD, YZh, and YC provided the experimental technical support. LZ provided guidance for the experimental idea. NL provided help in grammar modification. NT and ZQ are corresponding authors and provided guidance for the entire experiment and the writing of the article. All authors contributed to the article and approved the submitted version.

## Funding

This work was supported by the National Natural Science Foundation of China (81630068, 31670881 to ZQ).

## Conflict of Interest

The authors declare that the research was conducted in the absence of any commercial or financial relationships that could be construed as a potential conflict of interest.

## Supplementary Material

Supplementary Figure 1 MEFs were transfected with hTERT retrovirus containing a puromycin-resistance gene. Immortalized MEFs with puromycin-resistance were co-injected with CT-26 cells to build tumor bearing mouse model. The mouse was sacrificed and tumor was dissociated after 10 days growth. Puromycin (2 μg/ml) were used to select CAFs by killing cells without puromycin-resistance.
