# Supplementary figures and images for: Polysaccharides From Lentinus Edodes Inhibits Lymphangiogenesis via the Toll-Like Receptor 4/JNK Pathway of Cancer-Associated Fibroblasts
Source: Front Oncol. 2021 Feb 12;10:547683. doi: 10.3389/fonc.2020.547683 (PMC7907162; doi:10.3389/fonc.2020.547683)

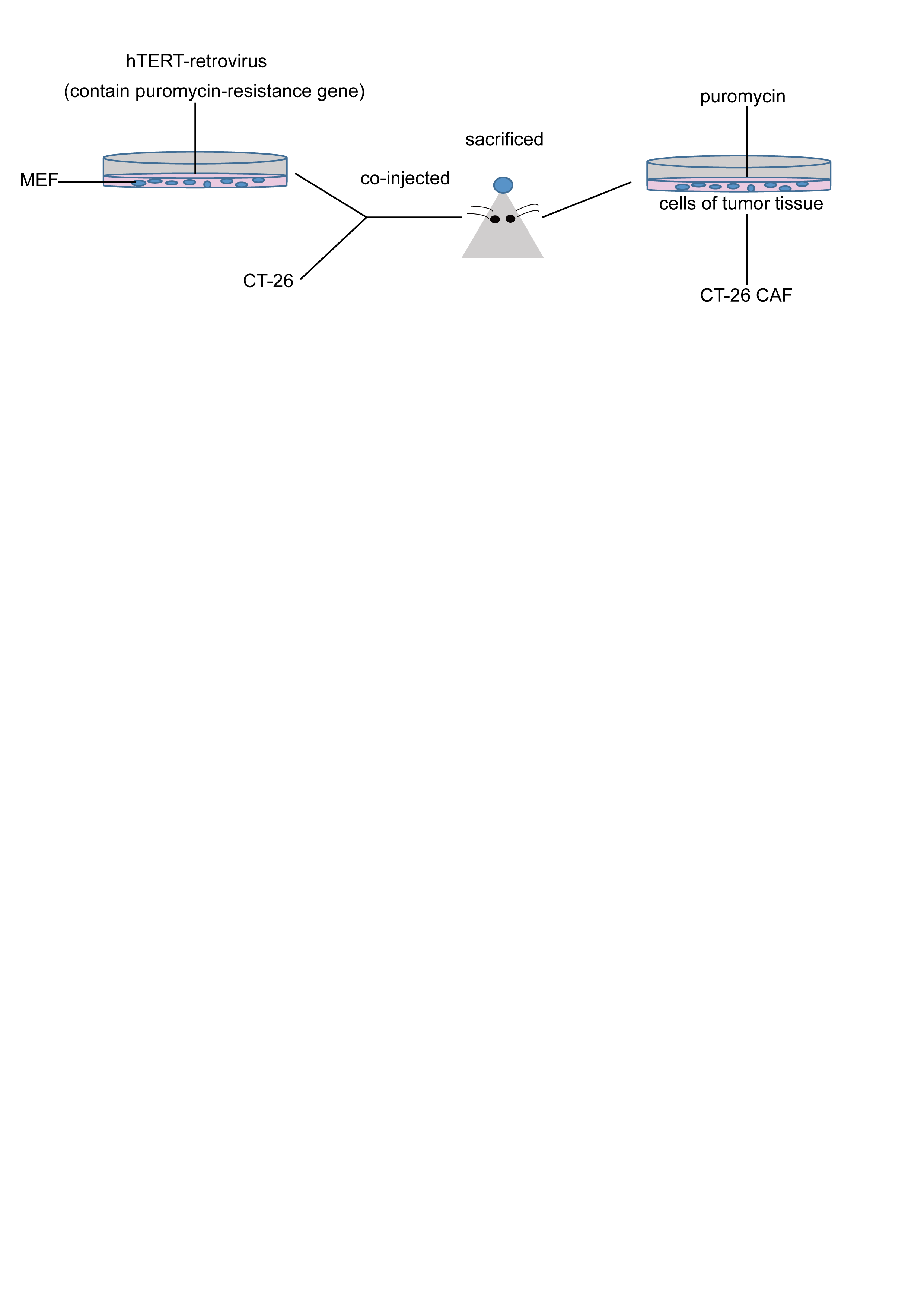

Supplement: Supplementary Figure 1 — MEFs were transfected with hTERT retrovirus containing a puromycin-resistance gene. Immortalized MEFs with puromycin-resistance were co-injected with CT-26 cells to build tumor bearing mouse model. The mouse was sacrificed and tumor was dissociated after 10 days growth. Puromycin (2 μg/ml) were used to select CAFs by killing cells without puromycin-resistance. [file Image_1.tif]
